# Assessment of CAD/CAM Customized V Pattern Plate Versus Standard Miniplates Fixation in Mandibular Angle Fracture (Randomized Clinical Trial)

**DOI:** 10.1007/s12663-023-02027-x

**Published:** 2023-10-27

**Authors:** Hiba Obad Saleh, Basma Gamal Moussa, Khaled Amr Salah Eddin, Samer Abduljabar Noman, Ahmed Mohammed Salah

**Affiliations:** 1 Department Oral & Maxillofacial Surgery, Faculty of Dentistry, Cairo University, Cairo, Egypt; 2 Department of Oral & Maxillofacial Surgery, Faculty of Dentistry, Sana’a University, Sana’a, Yemen; 3 Department of Oral & Maxillofacial Surgery, Ahmed Maher Teaching Hospital, Cairo, Egypt

**Keywords:** Customized plate, Virtual planning, Mandibular angle fracture, Mimics’ software, Postoperative complication, Superior–inferior plate

## Abstract

**Background:**

Mandibular angle is the most common site for fractures, accounting for 23–42% of all cases of mandibular fractures. A customized fixation system is designed directly for a specific patient, which reduces the time spent bending and fixing the plate during the operation. This study was designed to assess the effect of CAD/CAM customized V pattern plate versus standard miniplates fixation in mandibular angle fracture.

**Materials and Methods:**

This prospective randomized clinical trial included 26 patients suffering from mandibular angle fracture. Patients were selected from Oral and Maxillofacial Surgery Department, Faculty of Dentistry, Cairo University and Ahmed Maher Teaching Hospital. Study group (13) needed open reduction and internal fixation by using CAD/CAM V plate with surgical guide, while control group (13) needed open reduction and internal fixation by using standard superior–inferior miniplate fixation. The patients were then followed up for one year postoperatively.

**Results:**

It showed that there was a statistical difference between the study group and the control group regarding postoperative pain, occlusion, and maximal interincisal opening (*p* value < 0.05%). There was no statistical difference (*p* value > 0.05%) in the postoperative panoramic radiograph that was taken within the postoperative 1st week in both groups, while the increase in mean bone density was statistically significant (*p* value < 0.05%) from 6 months to one year postoperatively.

**Conclusion:**

CAD/CAM customized V pattern plate is a suitable plate design because it offers sufficient stability for normal bone healing, the creation of an ideal occlusion, an early return to function, and adequate postoperative radiographic outcomes.

**Trial Registration:**

It was registered at ClinicalTrials.gov. Registration number: NCT03761524. Registration date: 03.12.2018.

## Background

Mandibular angle is a highly weak bone as it is located at the junction of the ramus and the lower body of the mandible so it is readily shattered in violent crimes, sports, auto accidents, or pathological processes. Mandibular Angle Fracture is defined as a fracture line running inferiorly across the inferior border or posteriorly toward the gonial angle, starting where the anterior border of the mandibular ramus meets the body of the mandible [1–4].

Among all mandibular fractures, mandibular angle fractures have the highest postoperative complications [5]. The most common form of management for mandibular angle fractures is the use of one miniplate according to Champy guidelines in 1975. Champy suggested placing a single non-compressive miniplate on the mandibular superior border, which corresponds to the mandibular tension band (Champy technique) [6–9]. In certain constellations, two miniplates with superior miniplate fixed in tension zone and inferior miniplate fixed in compression zone were used. However, based on recent experimental and clinical studies, the stability provided by the miniplates fixation of mandibular angle fractures has become a point of contention among surgeons [10, 11].

In order to decrease the need for plate removal that may result from postoperative complications, reduce the operative time, and improve the stability of the fixation system, CAD/CAM customized plate design is using the minimum output values for stress of plate and displacement of bony segments to seek maximum reduction volume on an original plate [7, 8].

When compared to standard miniplates, a customized fixation mechanism is considerably more suited to the bone surface, which will shorten the time needed to bend and fix the plate during surgery [8]. It makes it possible for a plate to adapt to a bone easily and without distortion, which helps to meet the requirements of a semirigid fixation with less complications [12]. The aim of this study was to clinically and radiographically evaluate the effectiveness of using CAD/CAM customized V pattern plate fixation versus standard superior–inferior miniplates fixation in mandibular angle fracture.

## Materials and Methods

### Study Design and Participants

This randomized clinical trial was designed according to the CONSORT checklist and conducted at the Faculty of Dentistry at Cairo University and Ahmed Maher Teaching Hospital in Egypt from January 2019 to March 2022. A total of 26 patients with unilateral unfavorable mandibular angle fractures were randomized in equal proportions between two groups; a study group (*n *= 13) was treated with open reduction and internal fixation by using CAD/CAM customized V pattern plate fixation. Customized V pattern plate fixation was guided by the surgical guide, and a control group (*n *= 13) was treated with open reduction and internal fixation by the standard superior–inferior miniplates plate fixation. Both surgical procedures were performed by the same chief surgeon.

### Eligibility Criteria

#### Inclusion Criteria

Twenty-six dentate adult patients with a unilateral unfavorable angle fracture associated with ipsilateral body fracture were included in this study. All patients did not have any missing teeth. We included patients with unilateral unfavorable angle fractures associated with ipsilateral body fractures due to the limited number of isolated angle fracture cases in our department. 26 patients were randomly assigned into two identical groups each of which was 13 patients by using special website concerned with randomization process called Researcher Randomizer (https://www.randomizer.org/).

#### Exclusion Criteria

Patients with systemic disease and patients who cannot tolerate follow-up intervals (which affect the accuracy of study results).

### Preoperative Virtual Planning and Surgical Simulation Phase

**Fig. 1:**
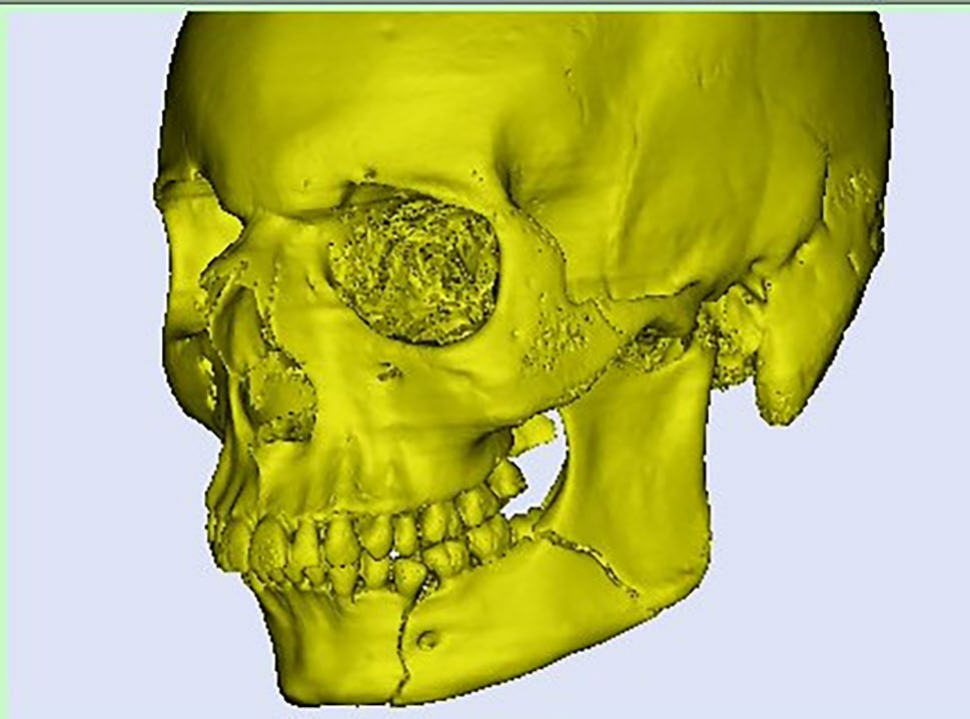
Creation of 3D model of the skull and mandible after segmentation 2

**Fig. 2:**
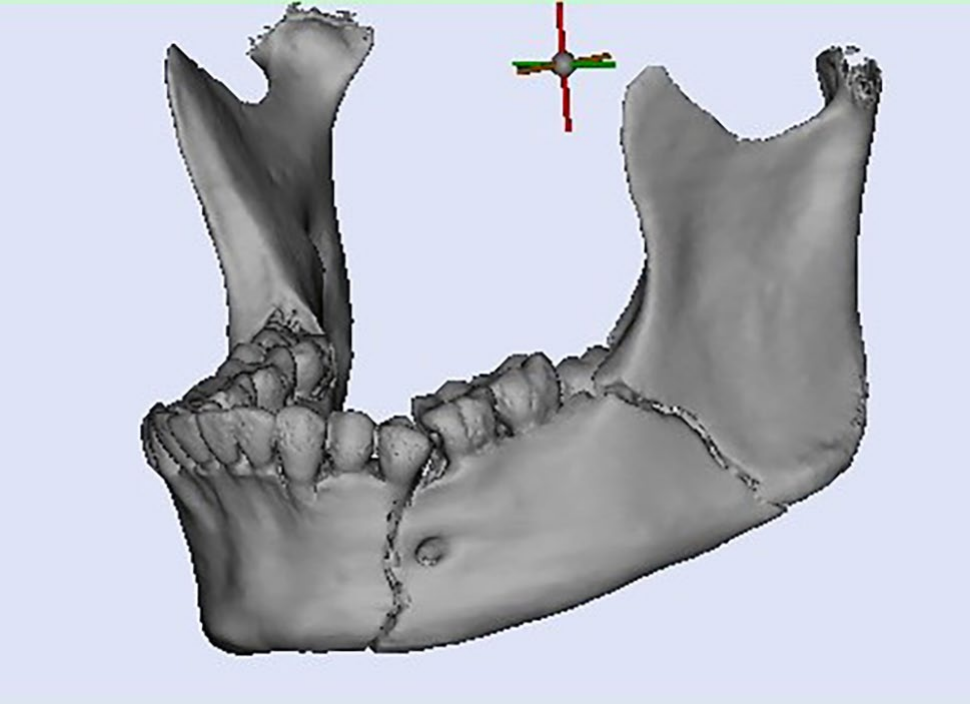
Separation and isolation of the mandible from the cranium 2

**Fig. 3:**
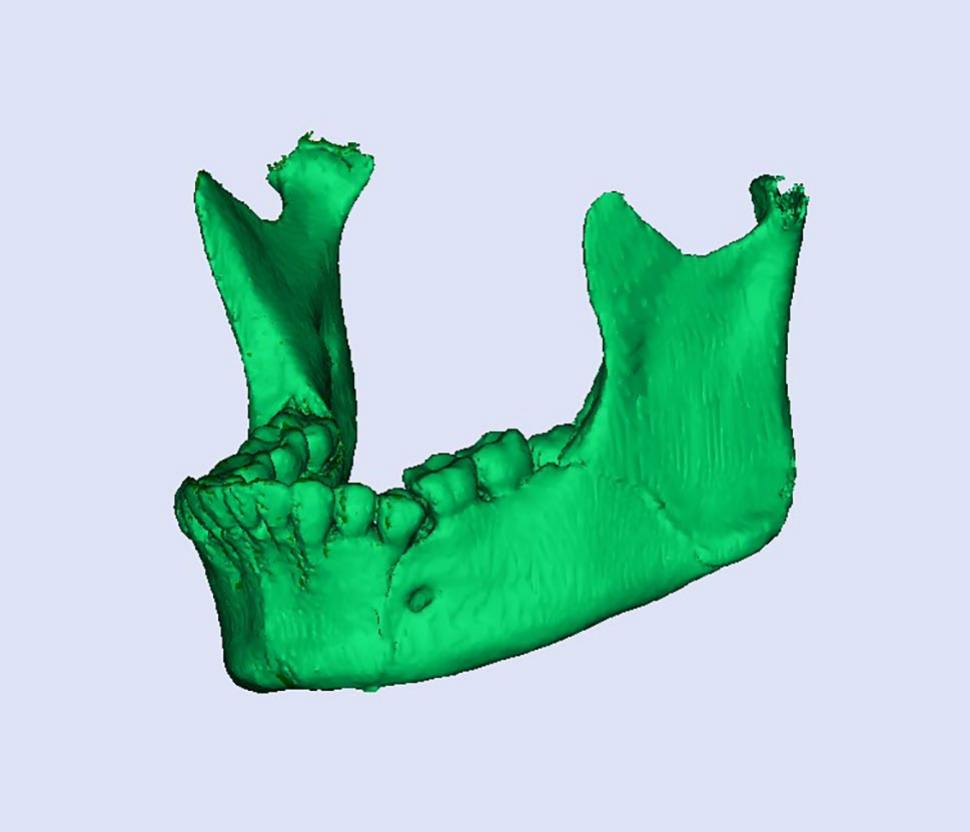
Virtual reduction of the fractured angle segments and reunion of both segments according to the adopted protocol in study group 2

**Fig. 4:**
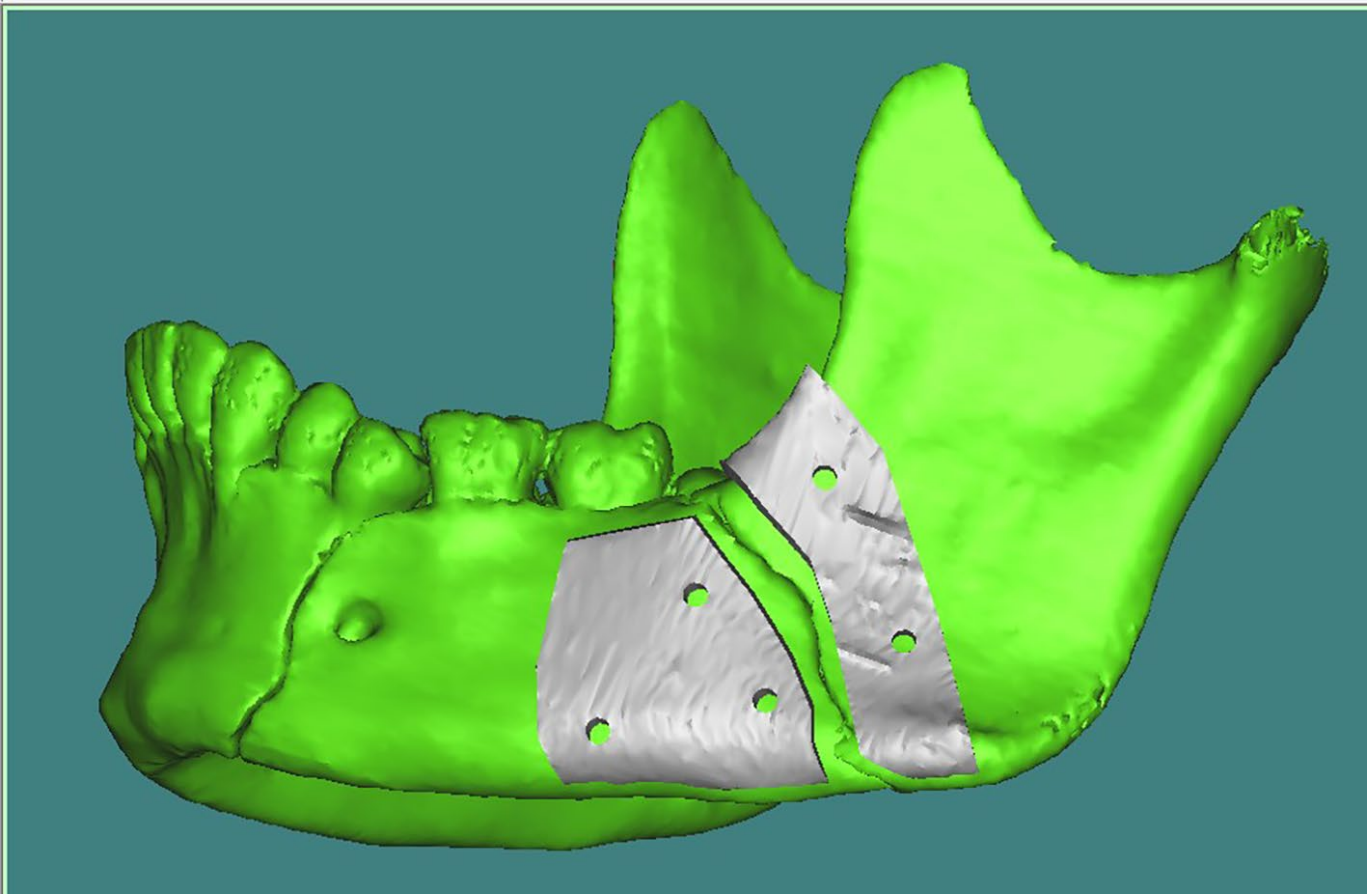
Surgical guide 2

**Fig. 5:**
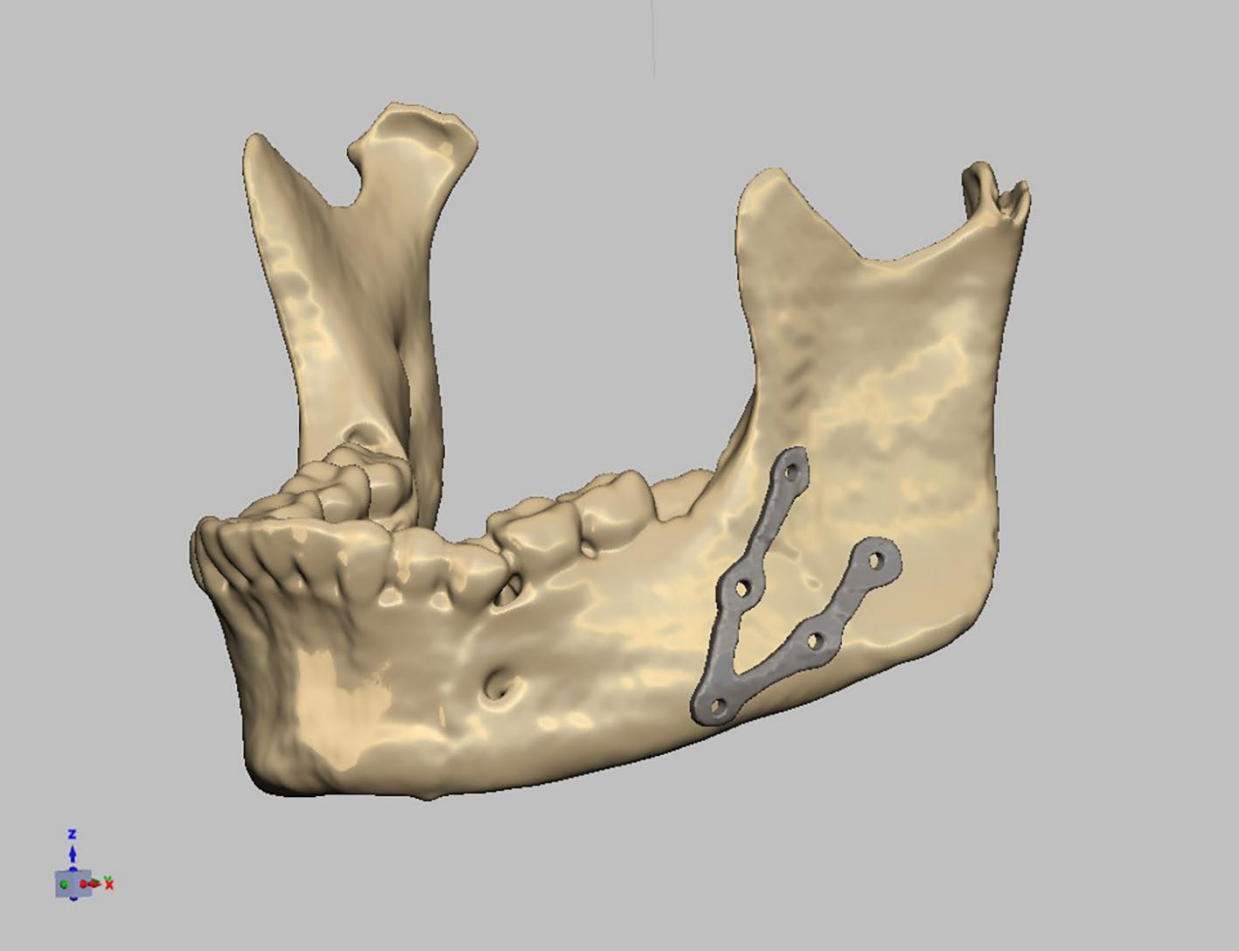
Preplanned mandibular reduction with plate design 2

**Fig. 6:**
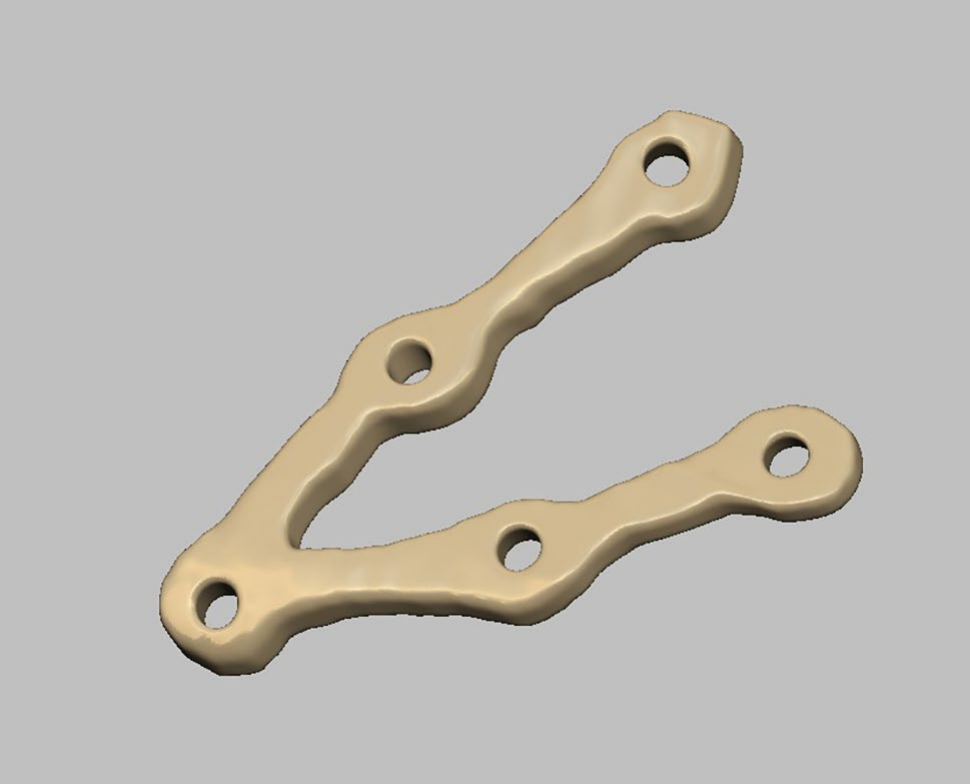
Customized V pattern plate 2

**Fig. 7:**
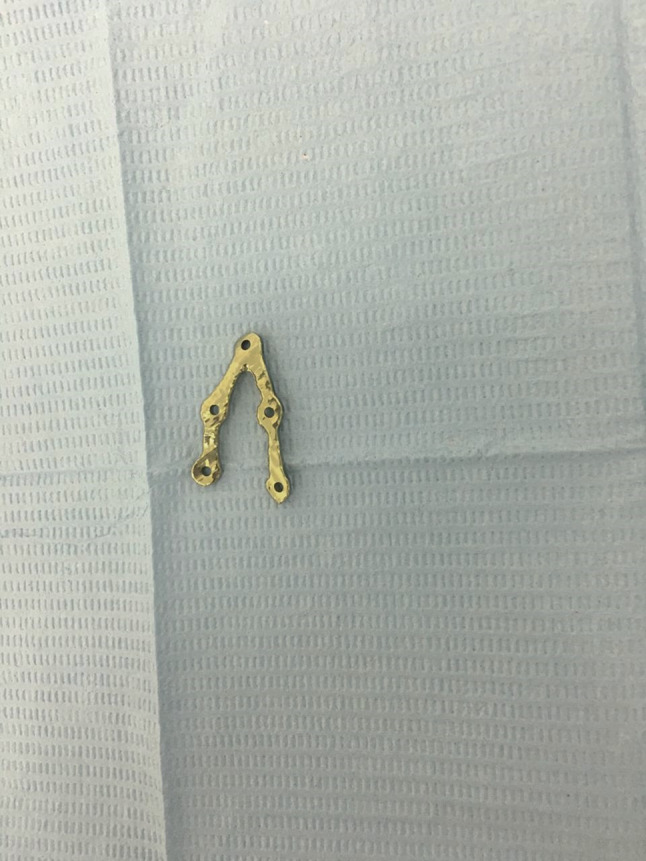
V plate design after milling 2

Planning was done using Mimics Innovation Suite 15.0 software for surgical planning, surgical simulation, and modeling of a custom-made V pattern plate, which was installed on a standard personal computer system. The developments consisted of a given CT scan and included the main image processing steps of a 3D computer model of the entire skull, a standard image orientation, mandible separation from the cranium, fracture segmentation, and virtual fracture reduction in Figs. 1, 2, and 3. When the desired surgical guide was completed, it contained two segments and five holes of 2 mm thickness, which corresponded to the holes of the V plate. The first segment had three holes of 2 mm thickness and was placed distal to the fracture line, and the second segment had two screw holes and was placed proximally to the fracture line. This guide helps ensure accurate placement of the screw holes and the V plate path away from any vital structures (teeth apices and inferior alveolar nerve) in Fig. 4. The construction of the plate is a V shape (2–2.3 mm thickness) to align the fractured segments together with 2 arms on either side of the fracture line and five holes for 2 mm screws away from the tooth apices and inferior alveolar nerve. The distances between screw holes in a customized plate can be changed for different patients, especially for patients with bad bone conditions in Figs. 5, 6. The milling of a plate design on a titanium block was done by a CAM system in Fig. 7.

### Surgical Technique Phase

The operation was performed under general anesthesia. A mandibular vestibular incision was placed 3–5 mm inferior to the mucogingival junction, leaving unattached mucosa on the alveolus to facilitate the closure. The fracture was exposed by elevating a mucoperiosteal flap down to the inferior border of the mandible. The fractured segments were mobilized and aligned to obtain proper reduction, and then, maxillomandibular fixation was applied to obtain appropriate occlusion in both groups as all patients had adequate number of teeth for correct maxillomandibular fixation.

**Fig. 8:**
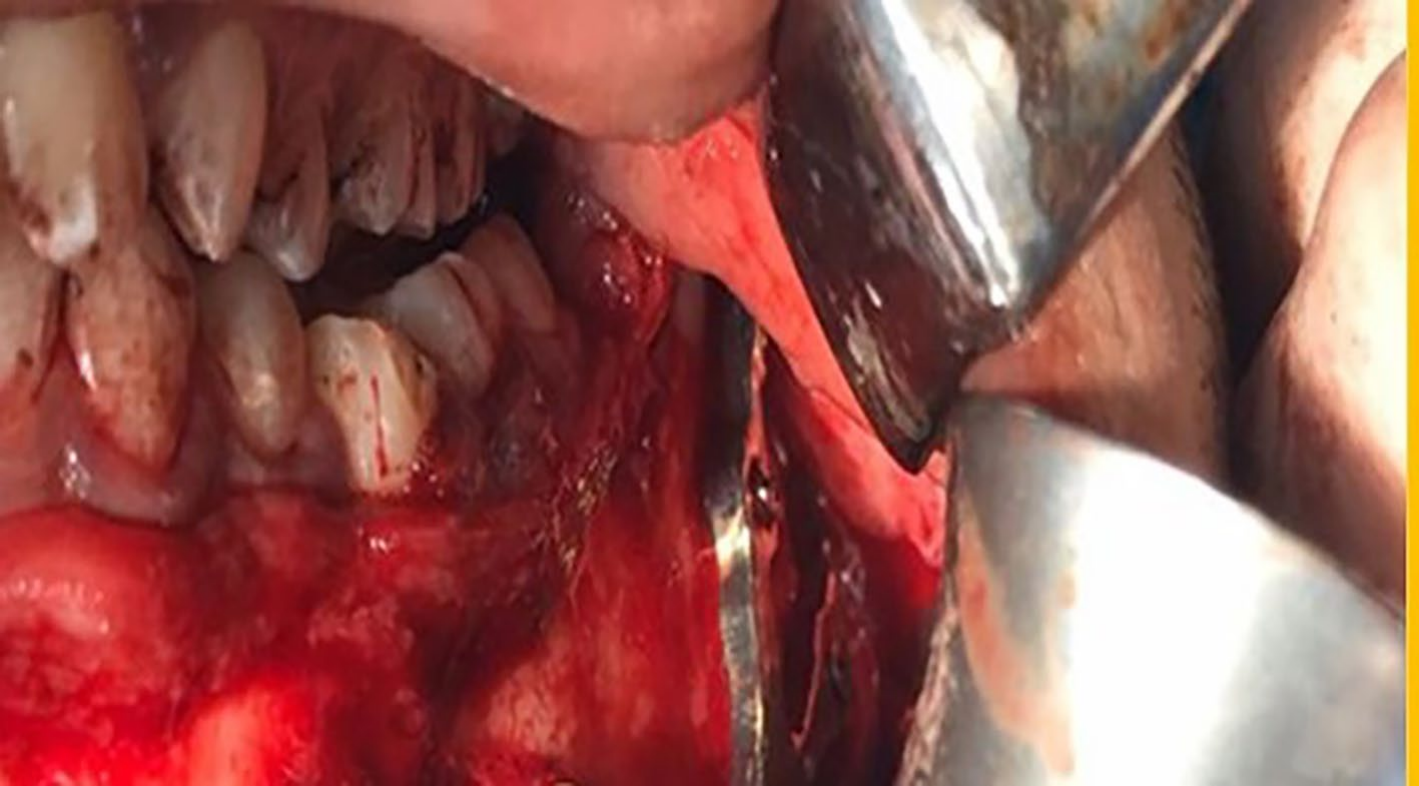
Customized V pattern plate after fixation in study group 3

*Fixation of the angle fracture in study group* was achieved by applying a V-shaped customized plate using a surgical guide that contained two segments and five holes of 2 mm thickness that corresponded to the holes of the V-shaped plate. The first segment had three holes of 2 mm thickness and was placed distal to the fracture line, and the second segment had two screw holes and was placed proximally to the fracture line. This guide helped to accurately place the screw holes and the V plate path away from any vital structures (teeth apices and inferior alveolar nerve). After detection of the correct position of the plate, V plate with its two arms was seated over the fracture site and adapted along the outer cortex of the mandible by using a trans-buccal trocar. The holes were drilled using a 2 mm diameter drill under copious irrigation with normal saline as a coolant. Then, a custom-made plate was secured with monocortical screws (6–8 mm) in the superior arm, while in the inferior arm bicortical screws (12–13 mm) were used. The bicortical screws were used for fixation of lower arm of V plate as it was located in the compression zone in Fig. 8.

**Fig. 9:**
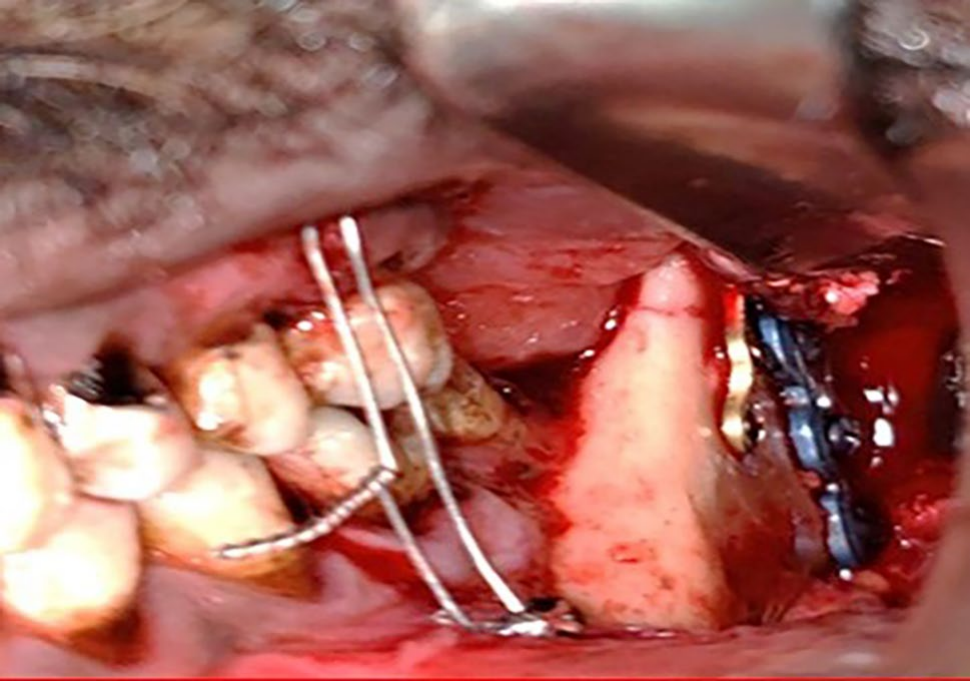
Standard superior–inferior miniplate fixation in control group 3

*Fixation of the angle fracture in the control group* was done by using superior and inferior miniplates fixation in order to provide a more stable fixation for mandibular angle fractures. It is generally accepted that during function of the lower jaw, tension will occur at the level of the dentition, whereas an effect of compression will be observed along the lower border. The superior plate (2.0 mm) was placed on the external oblique ridge or just lateral to it and fixated by monocortical 2 mm thickness screws (5–7 mm length), and the inferior plate (2.3 mm) was placed above the inferior border of the mandible and fixated by bicortical 2 mm thickness screws (12–13 mm length) The bicortical screws were used for fixation of inferior miniplate as it was located in the compression zone. The advantage of using bicortical screws was to avoid the effect of compression that it would be observed in the lower jaw, while its disadvantage was the possibility of inferior alveolar nerve injury in Fig. 9. The associated fracture beside the angle fracture was managed with open reduction and internal fixation.

After fixation of the fracture in both groups, the stability of the fixation was checked, MMF was removed and the occlusion was checked. Then, the wound was sutured back using continuous suturing with a 4-vicryl suture.

### Outcome Evaluation Phase

#### Postoperative Clinical Evaluation (Primary Outcome) Included

*Pain* was measured on the visual analogue scale (VAS). The patients were asked to rate their postoperative pain on a 4-point scale (0 = none, 1 = mild, 2 = moderate, and 3 = severe). Pain was recorded during the postoperative first and second weeks.

*Occlusal relationship* was checked in the maximal intercuspal position (centric occlusion) to ensure proper occlusal relationship, including molar relation and midline centralization. Any occlusal disturbance, including an open bite or improper tooth contact, was noted. This occurred immediately after the procedure and in the postoperative first week, one month, three months, and six months postoperatively.

*The maximal interincisal opening* measurement was obtained by measuring the maximal interincisal opening between maxillary and mandibular central incisors during the postoperative first week, first month, three months, and six months.

### Postoperative Radiographical Evaluation (Secondary Outcome) Included

*Orthopantomogram (OPG)* was taken during the postoperative first week to assess adequate reduction of the fractured segments.

*Postoperative computed tomography (CT)* was obtained during the first six months and one year postoperatively. These images were imported into Blue Sky Plan software 4, where sagittal, axial, tangential, and virtual 3D CTs were produced. Radiographically, bone healing at the fracture site was observed, and careful attention was paid to the presence of infection and hardware failure (plate fracture or screw loosening). Radiographic bone density in Hounsfield units (HU) was measured at the fracture site.

*Sample size* was estimated to be 26 patients. Level of significance: 0.05%, effect size used in calculation: *d *= 0.9707, power of the study: 0.84, and the statistical test used is Cohen’s D.

*Statistical Analysis* Statistical analysis was performed with IBM^®^ SPSS^®^ (SPSS Inc., IBM Corporation, NY, USA) Statistics Version 26 for Windows.

## Results

### Patients’ Demographic Data

A total of 26 patients with unilateral unfavorable mandibular angle fractures were randomized in equal proportions between two groups; a study group (*n *= 13) was treated with open reduction and internal fixation by using CAD/CAM. Customized V pattern plate fixation was guided by the surgical guide, and a control group (*n *= 13) was treated with open reduction and internal fixation by the superior–inferior miniplates plate fixation. The mean age of patients was 28.19 years old. Most patients were male (69%), while only 31% were female.

### Postoperative Primary Outcomes Evaluation:

#### Pain Intensity

Results showed that pain intensity decreased from the first week to the second week in the study group undergoing open reduction and internal fixation by using CAD/CAM customized V pattern plate fixation guided by the surgical guide, with a mean of (2.77 ± 0.725, 1.46 ± 0.519), compared to patients in the control group who were undergoing open reduction and internal fixation by the superior–inferior miniplates fixation with a mean of (3.00 ± 0.707, 2.31 ± 1.032). There was a significant difference between the two groups (*P* < 0.05%). All patients in study group and control group had the same type of fracture which was unilateral unfavorable angle fracture associated with ipsilateral body fracture in Table 1.

**Table 1:** Pain intensity during postoperative first and second week

	Study group				Control group			
Postoperative pain score	First week		Second week		First week		Second week	
	Number	%	Number	%	Number	%	Number	%
No pain	0	0.00	7	70.0	0	0.00	3	30.0
Mild Pain	5	50.0	6	60.0	3	30.0	5	50.0
Moderate Pain	6	60.0	0	0.00	7	70.0	3	30.0
Sever Pain	2	20.0	0	0.00	3	30.0	2	20.0
Mean ± SD	2.77 ± 0.725		1.46 ± 0.519		3.00 ± 0.707		2.31 ± 1.032	
*P* value	*P* < 0.05%				*P* < 0.05%			

#### Occlusion

All patients did not have any loss of teeth during trauma. All patients had adequate number of teeth for correct maxillomandibular fixation. There was statistically significant difference between two groups with a *p* value  < 0.05%. A satisfactory level of occlusion was achieved in 42% of the study group and was maintained during the follow-up period. Only 2 patients (8%) in the study group had a mild discrepancy. On the other hand, satisfactory occlusion was achieved in 31% of the control group, and 3 patients (12%) had mild discrepancies and 2 patients (8%) had moderate discrepancies (Table 2).

**Table 2:** Study and control group * Postoperative occlusion cross tabulation

	Postoperative occlusion			Total	*P* value
	Satisfactory	Mild discrepancy	Moderate discrepancy		
Study group	11	2	0	13	0.05
	42%	8%	0.0%	50%	
Control group	8	3	2	13	
	31%	11%	8%	50%	
Total	19	5	2	26	
	73%	19%	8%	100%	

#### Maximal Interincisal Opening

There was a statistically significant difference regarding MIO in both groups in the postoperative first week and first month; however, there was no statistically significant difference during the postoperative third and sixth months.

#### In the Study Group

Preoperative MIO mean ± SD was (20 mm ± 2.9), in the first postoperative week, patients still had limited mouth opening mean ± SD (25 mm ± 5.3), by following up with the patients during the postoperative first month, MIO increased to become nearly normal (37 mm ± 1.6), and in the postoperative third and sixth months, MIO became normal (39 mm ± 3.3, 41 mm ± 2).

#### In the Control Group

Preoperative MIO mean ± SD was (19 mm ± 6.4), in the first postoperative week, patients still had limited mouth opening. (21 mm ± 2.6), by following up with the patients during the postoperative first month, MIO gradually increased to become (32 mm ± 3.3), and in the postoperative third and sixth months, MIO became normal. (39.1 mm ± 3.4, 40 mm ± 2.7) in Table 3.

**Table 3:** Maximal mouth opening of study group versus control group at different observation periods

Observation period	Study and control group	Mean (mm)	Std deviation	*P* value
Preoperative mmo	Study group	20	2.9	0.05
	Control group	19	6.4	
One-week postoperative mmo	Study group	25	5.3	0.035
	Control group	21	2.6	
One-month postoperative mmo	Study group	37	1.6	0.05
	Control group	32	3.3	
Three-month postoperative mmo	Study group	39	3.3	0.001
	Control group	39	3.4	
Six-month postoperative mmo	Study group	41	2.3	0.05
	Control group	40	2.7	

### Postoperative Secondary Outcomes Evaluation

#### Postoperative Panoramic Radiographs

Postoperative panoramic radiographs that were taken within the postoperative 1st week showed a good alignment of the osseous borders of the fracture line (12 patients in the study group and 11 patients in the control group) with good union of the osseous borders of the mandible and continuity of the inferior alveolar canal when crossed by the fracture, while inadequate alignment showed in one patient in the study group and two patients in the control group). There was no statistically significant difference in both groups with the *p* value > 0.05% in Table 4.

**Table 4:** One-week postoperative OPG

	Study and control group	One-week postoperative OPG		Total	Mean	Std. deviation	*P* value
		Adequate	Inadequate				
Postoperative OPG	Study group	12	1	13	1.08	.277	0.235
		46%	4%	50%			
	control group	11	2	13	1.15	.376	
		42%	8%	50%			
	Total	88%	12%	100%			

#### The Mean Bone Density

**Fig. 10:**
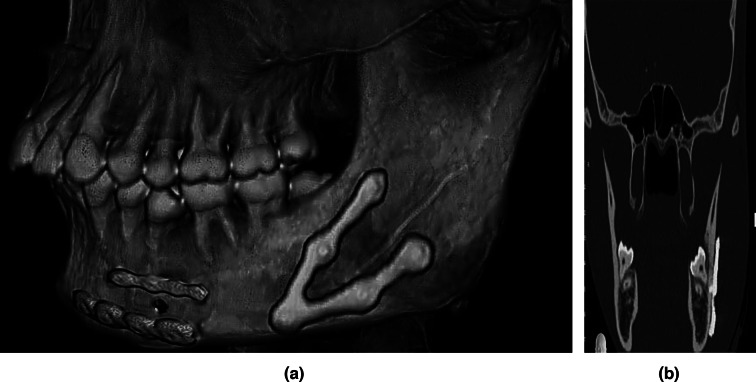
a, b Postoperative one-year coronal and 3D views revealing good adaptation of the custom-made V plate (Study group) 5

In the study group, the increase in mean bone density was statistically significant from 6 months (1637 HU) to one-year follow-up (1730 HU) (*p* value < 0.05%). In control group, there was an increase in bone density from 6-month (1071 HU) to one-year follow-up (1238HU) in Fig. 10a, b, Table 5.

**Table 5:** Bone density of study group versus control group at different observation periods

	Study and control group	Mean	Std. deviation	*P* value
6-month postoperative bone density	Study group	1637 HU	198.6	0.05
	Control group	1071 HU	153.8	
One-year postoperative bone density	Study group	1730 HU	170.6	0.05
	Control group	1238 HU	194.8	

## Discussion

Mandibular angle fracture management is difficult as there is no general standard protocol to treat angle fractures. Several types of miniplate fixations have been designed that are superior to other types in terms of stability and have fewer complications [13]. Semirigid internal fixation requires little or no maxillomandibular fixation, thus allowing earlier physical rehabilitation and function for the patient [14, 15]. A customized plate has some advantages; it has an easy application as well as simultaneous stabilization at both the superior and inferior borders [16]. This study was conducted to clinically and radiographically evaluate the effectiveness of using CAD/CAM customized V pattern plate fixation versus conventional superior–inferior miniplate fixation in mandibular angle fracture.

*In this study*, 26 patients with unilateral unfavorable mandibular angle fractures were randomized in equal proportions between two groups; a study group (*n *= 13) was treated with open reduction and internal fixation by using CAD/CAM. Customized V pattern plate fixation was guided by the surgical guide, and a control group (*n *= 13) was treated with open reduction and internal fixation by the conventional superior–inferior miniplates plate fixation.

*Regarding postoperative pain* was measured on the visual analogue scale (VAS), and pain intensity decreased from the postoperative 1st week to the 2nd week in the study group undergoing open reduction and internal fixation by using CAD/CAM. Customized V pattern plate fixation is guided by using the surgical guide with a mean of (2.77 ± 0.725, 1.46 ± 0.519). Decreased pain intensity rates in the study group could be attributed to decreased operative time because of the correct design of the CAD/CAM customized V pattern plate and its direct application in the correct place that was guided by the cutting surgical guide. These results were in agreement with other studies that reported that the decreased infection rate in 3D miniplate studies versus 2 miniplate studies may be attributable to the low plate profile, which means less hardware, and to decreased operative time because of the malleability of the 3D plate and direct application of the upper and lower struts of the 3D plate [17, 18].

*In the present study, there was a statistical difference (p* < *0.05%) in postoperative occlusion between study group and control group*. In 42% of the study groups, satisfactory occlusion was achieved and was maintained during the follow-up period. Only 8% of the patients in the study group had a minor discrepancy, but by the fourth postoperative week, they had all achieved satisfactory occlusion. This is consistent with the literature in which clinical studies investigating 3-dimensional plates showed that the occurrence of occlusal changes ranged from 0 to 6%, which is most probably a result of the higher stability of fracture segments and consequently stable occlusion offered by the 3-dimensional plate design [19–21]. On the other hand, satisfactory occlusion was achieved in 31% of the control group, 11% had a mild discrepancy, and 8% had a moderate discrepancy. This occlusal derangement could be the result of inadequate fracture reduction and fixation of the inferior miniplate. These results were in agreement with other studies. They found that the malocclusion varied from 0.5 to 7.8% in mandibular angle fractures that were treated with conventional miniplate fixation [22, 23].

*The results of this study showed that there was statistically significant difference in maximal interincisal opening in both groups in the preoperative, postoperative 1st week, and after one month.* The preoperative MIO mean in the study group was 20 mm. Patients had limited interincisal opening (25 mm) in the first postoperative week, but with follow-up during the first postoperative month, MIO increased to nearly normal (37 mm). This quick achievement of MIO occurred due to correct virtual planning and fixation of the plate in a short time that led to a decrease in postoperative edema and good improvement in MIO. In the control group, the preoperative MIO mean was 19 mm, and in the postoperative 1st week, patients still had limited mouth opening (21 mm). By following up with the patients during the postoperative 1st month, MIO gradually increased to 32 mm. However, there was no statistically significant amount of MIO (*p* value was > 0.05%) in the postoperative 3rd and 6th months in both groups, MIO in the study group became normal (39 mm, 41 mm), and in the postoperative 3rd and 6th months, MIO in the control group became normal (39 mm, 40 mm). Our results were similar to the results obtained by three other studies. They found that the mean maximal mouth opening in their study was less than 30 mm at immediate postoperative and 1st-week follow-ups, and more than 30 mm at 1st- and 3rd-month follow-up [24, 25].

*In the present study, there was no statistically significant difference in postoperative OPG in both groups (p* > *0.05%).* Only one patient in the study group and two patients in the control group had an inadequate alignment of the fracture borders. These results were in agreement with other studies that addressed the possibility that nonunion or malunion may be due to the enrollment of patients with poor nutritional status, metabolic disturbances, or medically compromised states, all of which can lead to inadequate bone healing. Other local reasons may be related to infections at the fracture site, tissue or foreign bodies between the segments, comminution of the fracture, or inadequate union of the fracture segments [26, 27]. Other studies reported that proper bone union was observed in all patients with no cases of displacement or any step deformity [28, 29].

*In the current study, both groups showed an increase in postoperative bone density along the fracture line from 6 months to one year (p value* < *0.05%).* At 6-month postoperative follow-up, the bone density values in the study group were (1637 HU) and (1071 HU) in the control group. During the postoperative one-year follow-up, the bone density values were 1730 HU in the study group and 1238 HU in the control group. This study was conducted between CAD/CAM customized plate and standard miniplates fixation, and its results were in agreement with other studies [30, 31]. They found that their results were within the normal range, where the cortical bone density of the mandible ranged between 800 and 1580 HU at the alveolar bone and 1320 and 1560 HU at the basal bone. Cancellous bone in the mandible had densities of 300–500 HU for alveolar bone and 170–440 HU for basal bone. Another study [32] also concluded that the mean bone density after 12 weeks showed a statistically significant increase in its values when compared to the immediately postoperative values (*p* < 0.001).

Based on the results of our study, further studies with a larger sample size, a longer follow-up period, are recommended to be done to assess CAD/CAM customized V pattern plate versus other type of miniplate fixation for mandibular angle fracture to get more results.

## Conclusion

CAD/CAM customized V pattern plate is a suitable plate design because it offers sufficient stability for normal bone healing, the creation of an ideal occlusion, an early return to function, and adequate postoperative radiographic outcomes.

## Data Availability

The datasets used and/or analyzed during the current study are available from the corresponding author on reasonable request.

## Abbreviations

CT: Computed tomography
HU: Hounsfield unit
MIO: Maximal interincisal opening
OPG: Orthopantomogram
